# Insulin Clearance in Obesity and Type 2 Diabetes

**DOI:** 10.3390/ijms23020596

**Published:** 2022-01-06

**Authors:** Han-Chow E. Koh, Chao Cao, Bettina Mittendorfer

**Affiliations:** Center for Human Nutrition, Washington University School of Medicine, 660 S Euclid Ave, Campus Box 8031-14-0002, St. Louis, MO 63110, USA; hkoh@wustl.edu (H.-C.E.K.); caochao@wustl.edu (C.C.)

**Keywords:** insulin clearance, insulin extraction, insulin secretion

## Abstract

Plasma insulin clearance is an important determinant of plasma insulin concentration. In this review, we provide an overview of the factors that regulate insulin removal from plasma and discuss the interrelationships among plasma insulin clearance, excess adiposity, insulin sensitivity, and type 2 diabetes (T2D). We conclude with the perspective that the commonly observed lower insulin clearance rate in people with obesity, compared with lean people, is not a compensatory response to insulin resistance but occurs because insulin sensitivity and insulin clearance are mechanistically, directly linked. Furthermore, insulin clearance decreases postprandially because of the marked increase in insulin delivery to tissues that clear insulin. The commonly observed high postprandial insulin clearance in people with obesity and T2D likely results from the relatively low insulin secretion rate, not an impaired adaptation of tissues that clear insulin.

## 1. Introduction

### 1.1. Overview

Compared with healthy lean people, people with obesity have increased basal and postprandial plasma insulin concentrations [1,2,3]. People with obesity and type 2 diabetes (T2D) have lower postprandial insulin than those without T2D, and the relative insulin insufficiency is responsible for the marked hyperglycemia in people with T2D [1,2,3]. The prevailing thought is that the increase in plasma insulin in people with obesity is a compensatory response to obesity-associated insulin resistance. Presumably, pancreatic β cells and tissues that clear insulin sense the need to secrete more and clear less insulin to prevent hyperglycemia when there is insulin resistance, and this compensatory mechanism is impaired in people with T2D [4,5,6,7,8,9]. Here, we provide an overview of the factors that regulate insulin removal from plasma and discuss the interrelationships among plasma insulin clearance, excess adiposity, insulin sensitivity, and T2D in people with obesity. Collectively, the data in the literature we present in this review suggest that plasma insulin clearance is reduced in people with obesity who are insulin resistant, not because of compensatory adaptations in tissues that clear insulin, but simply because both insulin action in and insulin clearance by tissues require insulin binding to its receptors and insulin receptors are downregulated in people who are insulin resistant. Moreover, a marked increase in insulin delivery to tissues that clear insulin causes a decrease in plasma insulin clearance because insulin binding to its receptor causes endocytosis of the insulin–insulin receptor complex and a temporary decrease in insulin receptor density on the cell surface. So, the higher postprandial insulin clearance in people with T2D, compared to those without, is not a maladaptation in tissues that clear insulin, but simply a consequence of β-cell dysfunction and reduced postprandial insulin secretion, which blunts insulin delivery to tissues that clear insulin. This has important clinical implications because it suggests that pancreatic β cells, not tissues that clear insulin, are responsible for the higher postprandial plasma insulin clearance in people with T2D. Β cells, not tissues that clear insulin, should therefore be the primary treatment target aimed at decreasing postprandial insulin clearance.

### 1.2. Insulin Production and Delivery to Tissues That Take Up Insulin

**Figure 1:**
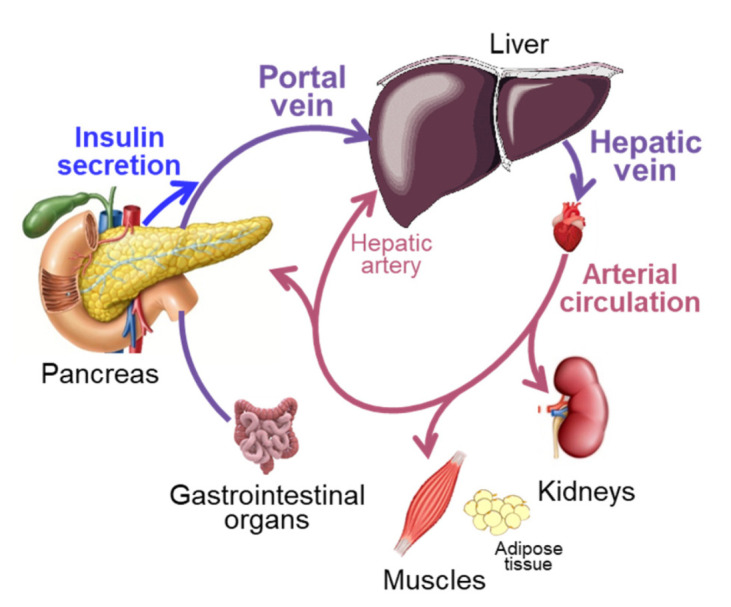
Insulin appearance in and removal from the circulation. Insulin is produced by pancreatic β cells and secreted into the portal vein, which delivers the newly produced insulin to the liver. The liver extracts approximately half of the newly secreted insulin. The remaining insulin is delivered to the systemic arterial circulation via the hepatic veins. Extrahepatic tissues extract some of the insulin from the arterial circulation during the first pass; the remaining insulin is removed in subsequent passes through the liver and extrahepatic tissues. Organ sizes in the figure are depicted according to their importance in determining plasma insulin concentration, rather than proportional to their actual size.

Insulin is produced by pancreatic β cells and secreted into the portal vein, which delivers the newly produced insulin to the liver. The liver extracts approximately half or more of the newly secreted insulin [10,11,12]; the remaining insulin leaves the liver via the hepatic veins and enters the systemic arterial circulation (Figure 1). Under basal conditions, the kidneys extract approximately 30–35% of the insulin that is delivered, and skeletal muscles and adipose tissue extract approximately 15% [12,13,14,15,16,17]. Insulin that is not extracted during the first pass through tissues is removed in subsequent passes. Total insulin delivery to the liver therefore represents the sum of insulin secretion and insulin delivery from the systemic circulation back to the liver through both the hepatic artery and the portal vein (after passage through the gastrointestinal tract) [18]. In total (first and subsequent passes), the liver takes up approximately 65% of the amount of insulin that has been secreted; the kidneys take up approximately 25% of the total amount secreted and skeletal muscles and other tissues and organs take up approximately 10%. The removal of insulin from the circulation is very efficient, because of the high extraction of insulin by the liver and kidneys. The mean residence time of insulin in the circulation is only a few (<10) minutes [19,20,21,22,23,24,25]. Nevertheless, very small differences in insulin clearance can have a major impact on plasma insulin concentration because the insulin secretion rate is very high in relation to the plasma insulin pool size [26]. Assuming average basal insulin secretion and clearance rates in people with obesity [8,27], a 10% decrease in plasma insulin clearance would cause a ~120 pmol/L (or ~20 mU/L) increase (approximately doubling of basal values) in plasma insulin concentration in just 10 min. Understanding the regulation of insulin removal from plasma and how it might be altered in people with obesity and T2D is therefore very important.

### 1.3. Cellular Mechanisms of Insulin Removal from Plasma

**Figure 2:**
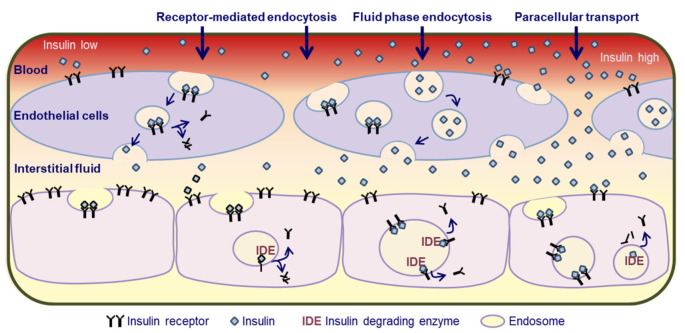
Insulin transport across the endothelium and cellular insulin uptake. Insulin must first pass through the endothelial cell layer of capillaries into the interstitium. The liver sinusoidal endothelial cell layer is fenestrated and highly permeable to insulin, allowing free flow of insulin towards hepatocytes. In skeletal muscles and adipose tissue, insulin crosses the tight endothelial cell layer via receptor-mediated endocytosis-exocytosis, fluid-phase transport, and paracellular transport. Receptor-mediated transcellular transport represents the key route of entry when blood insulin concentration is low and fluid-phase transcellular transport and paracellular transport take over when blood insulin concentration is high. Once in the interstitium, insulin binds to insulin receptors on parenchymal cells, which causes the translocation of the insulin–insulin receptor complex into the cytosol via endocytosis. Insulin is degraded inside the endosomes by insulin degrading enzyme and the receptors start to return to the cell surface.

For insulin to be taken up by the liver, skeletal muscles, and adipose tissue, it must first pass through the endothelial cell layer of capillaries into the interstitium (Figure 2). The liver sinusoidal endothelium is fenestrated and highly permeable to insulin, allowing free flow of insulin towards hepatocytes [28]. In skeletal muscles and adipose tissue, insulin crosses the tight endothelial cell layer via receptor-mediated endocytosis-exocytosis, fluid-phase transport, and paracellular transport [29,30,31]. The relative importance of these pathways for insulin entry into the interstitium depends on the blood insulin concentration; receptor-mediated transcellular transport represents the key route of entry when blood insulin concentration is low and fluid-phase transcellular transport and paracellular transport take over when blood insulin concentration is high [29]. The endothelial barrier causes a short delay in the appearance of insulin from the circulation in the interstitium [24,32,33]. Once in the interstitium, insulin binds to insulin receptors on parenchymal cells, which quickly (within minutes) causes the translocation of the insulin–insulin receptor complex into the cytosol via endocytosis [34,35,36,37,38,39,40] (Figure 2). In the liver, this process is mediated by carcinoembryonic antigen-related cell adhesion molecule 1 (CEACAM 1). CEACAM 1 is phosphorylated by the activated insulin receptor whereupon it binds to and traps the insulin–insulin receptor complex [34,41]. Once inside the cells, insulin is degraded within the endosomes by insulin degrading enzyme [34,41] (Figure 2). Some of the insulin is also degraded extracellularly while bound to insulin receptors at the plasma membrane [34] (Figure 2). In kidneys, most of the insulin is removed from plasma by glomerular filtration in addition to some peritubular uptake of insulin [42,43]. The filtered insulin is almost completely reabsorbed and degraded in cells lining the proximal convoluted tubules [42,43].

The rate and extent of insulin receptor endocytosis is directly related to the insulin concentration; the higher the insulin dose, the faster the internalization process and the greater the number of receptors that are trapped inside cells [38]. Once insulin has been removed from the receptor inside the cell, the “empty” receptors start to return to the cell surface and this recycling process takes upwards of 30 min [37,38,40,44]. Some of the receptors, however, are degraded in the cell and they can be replaced with newly synthesized receptors [40,45,46]. An acute increase in insulin exposure of cells therefore leads to a dose-dependent temporary loss of insulin receptors on the plasma membrane [47,48]. Insulin degradation in endosomes is primarily responsible for the dissociation of insulin from the insulin receptor; although, intact insulin is also released from receptors, and the rate of release of insulin from the receptor (via simple dissociation or degradation) is the key determinant of the rate of receptor recycling back to the plasma membrane [41,49,50,51,52]. In addition, the insulin–insulin receptor interaction itself depends on the dose and duration of exposure to insulin. The insulin receptor exists in two isoforms that are distinguished by the inclusion or exclusion of exon 11 in the mRNA [53,54,55]. The homologous insulin-like growth factor receptor also binds insulin [53,54,55]. The insulin and insulin-like growth factor receptors can form heterodimers that have different insulin binding affinities and exhibit negative cooperativity (i.e., reduced insulin binding at high compared with low doses of insulin) [53,54,55]. Some studies found pretreatment of cells with insulin in vitro can increase the affinity of the receptors for insulin [45,56,57,58,59]. However, it can also reduce insulin receptor signaling transduction [60,61]. Moderate experimental hyperinsulinemia in vivo, induced by insulin infusion, did not alter insulin binding or reduced it [62,63,64]. Insulin action, like insulin uptake, requires insulin binding to insulin receptors on the cell surface, and in some cases also endocytosis [41,65,66,67,68]. Insulin uptake into cells (plasma insulin clearance) and insulin action are therefore directly linked. The lower the receptor density and receptor affinity, the higher the dose of insulin that is required to bind to and activate a certain number of receptors.

### 1.4. Assessment of Insulin Removal from Plasma In Vivo: Units of Measurement

The removal of insulin from plasma in vivo is quantitated in multiple ways that provide unique and complementary insights into the insulin removal process. Plasma insulin clearance rate refers to the amount of plasma that is cleared of insulin per unit of time and is expressed as liters/minute. Insulin extraction or uptake rate refers to the molar amount of insulin that is removed from plasma per unit of time; it represents the product of insulin clearance rate and plasma insulin concentration and is expressed as pmol/minute. Therefore, the insulin extraction or uptake rate can be high even if the plasma insulin clearance rate is low, or vice versa, the extraction or uptake rate can be low even if the clearance rate is high [8]. Insulin fractional extraction refers to the fraction (typically expressed as percent) of the amount of insulin delivered that is taken up by a specific tissue or organ. Lastly, the insulin fractional catabolic rate provides an assessment of the turnover rate of the plasma insulin pool and is expressed as pools/minute, where pools refers to the total amount of insulin in the circulation (plasma insulin concentration × volume) [20,24,32]. The inverse of the fractional catabolic rate represents the mean residence time of insulin molecules in the circulation. These values can be used to derive the half-life of insulin in the circulation.

### 1.5. Effect of Insulin Dose on Insulin Removal by Tissues In Vivo

**Figure 3:**
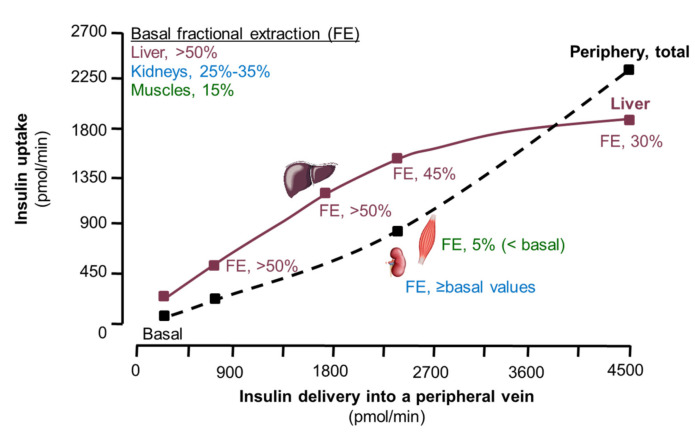
Relationship between insulin delivery and tissue insulin uptake and plasma clearance rate (adapted from references [12,13,14,15,16,72]). As insulin delivery to tissue increases, the fractional extraction of insulin (proportion of the amount delivered that is taken up) and plasma clearance of insulin decrease in the liver and skeletal muscles but increase in the kidneys. Furthermore, liver insulin uptake becomes saturated at approximately 1800 pmol/min whereas extra-hepatic insulin uptake is not saturable within the physiological range of insulin delivery to tissues. Note: arterial plasma insulin concentration in these experiments ranged from approximately 50 pM during basal conditions to approximately 9000 pM during the highest dose insulin infusion. Endogenous insulin secretion was inhibited by somatostatin infusion and insulin was infused into a peripheral vein. So, hepatic insulin delivery occurred almost exclusively via the arterial circulation, and hepatic insulin delivery at any arterial insulin concentration was, therefore, much less than during postprandial conditions when endogenous insulin secretion occurs into the portal vein. Abbreviations: Basal, overnight fasted condition; FE, fractional extraction.

Highly sophisticated studies that included arterio-venous blood sampling across various tissues and organs after administering insulin, or after stimulating endogenous insulin secretion, have demonstrated that the fractional extraction of insulin by tissues varies among tissues and is dependent on the insulin delivery rate [12,13,14,15,16,69,70,71] (Figure 3). During basal conditions, liver extracts >50% of the insulin that is delivered, kidneys extract ~30% and skeletal muscles extract approximately 15–35% [12,13,15,16]. When insulin delivery to the liver and muscles increases, insulin uptake (pmol/min) increases, but the fractional extraction of insulin by these tissues (percent delivered that is taken up) and plasma insulin clearance rate decrease [12,13]. Insulin clearance in the kidneys on the other hand is unaffected by increased insulin delivery or even increases with increasing insulin delivery [12,16], presumably because the bulk of insulin in the kidneys is removed by glomerular filtration and reabsorption via simple diffusion [42,43]. Increased renal insulin clearance can therefore compensate to some extent for the decrease in insulin clearance in liver and muscles.

The dose-dependent decrease in hepatic and muscle insulin clearance occurs within the postprandial insulin concentration range, but requires at least approximately a doubling or tripling of the basal insulin load. When arterial plasma insulin concentration was increased from approximately 50 pM to 300 pM by infusing insulin, insulin uptake (in pmol/min) by the forearm (muscle) increased, but the fractional extraction of insulin decreased from ~15% to ~5% [13]. Additionally, when insulin was administered into the portal vein as a slow bolus over 2.5 min, whole-body insulin clearance decreased markedly (up to 50%) as the dose of insulin increased from 5 mU/kg (~900 pmol/min) to 50 mU/kg (~9000 pmol/min) [69]. However, when increasing doses of insulin from 5 mU/kg to only 30 mU/kg were administered into a peripheral vein, resulting in lower maximal arterial insulin concentration and much lower hepatic insulin delivery rates (≤1500 pmol/min) than after portal insulin administration, whole-body plasma insulin clearance did not change [69]. When insulin was infused continuously into a peripheral vein, hepatic insulin clearance decreased when hepatic delivery of insulin increased by approximately >5-fold from basal values (to approximately 1800 pmol/min or more) [12]. Some studies that compared splanchnic insulin fractional extraction during basal conditions, when the liver receives newly secreted insulin from the pancreas and insulin from the arterial circulation, and during peripheral insulin infusion while endogenous insulin secretion was blocked by somatostatin infusion [14,72], found insulin extraction was unaffected by the insulin infusion [14,72]. The lack of suppression presumably occurred because hepatic delivery of insulin (<600 pmol/min) was not sufficiently increased. An acute, dose-dependent decrease in whole-body insulin clearance has also been observed when endogenous insulin secretion was stimulated by administering different doses of glucose intravenously [70] or orally [70,71,73] and insulin secretion rate at least doubled compared to basal values. In addition, prolonged (several hours) of exposure to high circulating insulin concentration (several-fold above basal values) reduced plasma insulin clearance [74,75].

Collectively, the results from the available studies [12,13,14,15,16,69,70,71,72,73,74,75] suggest that small increases (≤double) in whole-body and tissue insulin delivery above basal values have no effect on hepatic insulin extraction. However, large (several-fold) increases—in the order of magnitude observed postprandially [76,77]—reduce the fractional extraction of insulin in liver and skeletal muscles and regional and whole-body plasma insulin clearance (Figure 3). Furthermore, it was found that insulin uptake by the liver becomes saturated at approximately 1800 pmol/min (within the upper postprandial range) whereas extrahepatic tissues in totality can take up even more than 2500 pmol/min without signs of saturation [12]. The finite capacity for insulin removal by the liver can be explained by its size whereas the cellular mass of extrahepatic tissues is too large to become rate limiting. There are approximately 100 million hepatocytes per gram liver tissue [78] and results from radio-labeled insulin binding studies suggest that each of them can bind approximately 10,000–100,000 insulin molecules [79,80,81]. Assuming a liver weight of 1500 g [82], there are a total of 1.5 × 10^15^ to 1.0 × 10^16^ insulin binding sites per liver. If all of the binding sites were on the cell surface and occupied, we estimate the liver could bind at most upwards of 2500 pmol of insulin at once.

### 1.6. Study Protocols to Evaluate Plasma Insulin Clearance and Their Clinical Relevance

**Figure 4:**
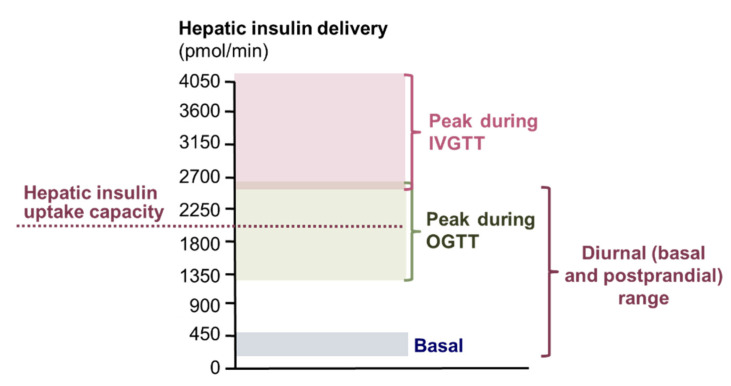
Hepatic insulin delivery during basal and postprandial conditions and during intravenous and oral glucose tolerance tests. Hepatic insulin delivery rates were calculated as described in reference [18] with average insulin secretion and plasma insulin concentration values reported in the literature. See text for details. Abbreviations: IVGTT, intravenous glucose tolerance test; OGTT, oral glucose tolerance test.

The paramount importance of the liver for the removal of endogenously produced insulin in combination with the complex regulation of cellular insulin uptake that is dependent on the insulin delivery rate has important implications for the interpretation of the results from studies that evaluated plasma insulin clearance in people with obesity and T2D. Plasma insulin clearance in people with obesity and T2D has been assessed by using an intravenous glucose tolerance test, an insulin-modified intravenous glucose tolerance test, an insulin suppression test, a hyperinsulinemic or hyperglycemic clamp procedure, an oral glucose tolerance test, and mixed meal tests [3,8,27,83,84,85,86,87,88,89,90,91,92,93,94,95,96]. The amounts and temporal dynamics of insulin appearance in the circulation differ markedly during these tests and are not always well suited to evaluate the normal diurnal regulation of insulin clearance (Figure 4).

Throughout the day, the insulin secretion rate from pancreatic β cells into the portal vein ranges from <200 pmol/min after an overnight fast in lean people to peak values of approximately 800 pmol/min postprandially in people with obesity and insulin resistance [76,77]. Arterial plasma insulin concentration is as low as <60 pmol/L in lean people after an overnight fast and can reach values of up to ~1000 pmol/L in people with obesity postprandially [76,77,97]. Total hepatic insulin delivery (insulin secretion rate plus insulin that is returned to the liver from the systemic circulation) ranges from <300 pmol/min to peak values of approximately 2000 pmol/min [18,95]. During oral glucose tolerance and meal tests, insulin secretion rate rises gradually during the first one to two hours and then starts to decrease towards basal values [3,8,95,98]. Insulin secretion and total hepatic insulin delivery rates during oral glucose challenge tests with up to 75 g of glucose [3,70,98,99] are similar to those observed after meal intake [76,77]. During an intravenous glucose tolerance test, on the other hand, the insulin secretion rate increases almost instantaneously to peak values (≥2000 pmol/min) [98,99,100,101] that far exceeds peak postprandial insulin secretion [76,77] and reaches or exceeds the hepatic capacity for insulin uptake [12] (see Section 1.5. Effect of insulin dose on insulin removal by tissues in vivo). The amount of glucose infused during a hyperglycemic clamp procedure can also elicit an insulin secretion rate and total hepatic insulin delivery rate (insulin secretion rate plus insulin that is returned to the liver from the systemic circulation) that well exceeds the hepatic capacity for insulin uptake [12,74,102].

The intravenous route of insulin delivery during some of the tests (hyperinsulinemic clamp procedure, insulin suppression test, insulin-modified intravenous glucose tolerance test), compared with the stimulation of endogenous insulin secretion, also requires consideration. Whole-body clearance of insulin that is administered into a peripheral vein is lower than whole-body clearance of the same amount of insulin appearing in the portal vein after intraportal insulin infusion or glucose-stimulated insulin secretion, because peripherally administered insulin is not subjected to hepatic first pass insulin extraction [18,103] (Figure 1). In addition, the relationship between arterial plasma insulin concentration and hepatic insulin delivery differs when insulin is delivered intravenously compared with being secreted by β cells. Although arterial insulin concentrations during hyperinsulinemic-euglycemic clamps are typically within the postprandial range (~600 pM), hepatic insulin delivery rates during hyperinsulinemic clamps are well below those observed postprandially [14,88,89,90,92,95,98,104,105,106] (Figure 3). Additionally, during an insulin-modified glucose tolerance test, the insulin dose that is administered intravenously results in arterial insulin concentrations that are similar to those after the initial glucose injection, so the insulin delivery to extrahepatic tissues is similar after glucose and insulin injection [98,100]. However, the hepatic insulin delivery rate (insulin secretion rate plus insulin that is returned to the liver from the systemic circulation) is markedly less after insulin injection than after glucose injection and does not quite reach the capacity for hepatic insulin uptake after insulin injection [98,100]. It has been proposed that incretins reduce plasma insulin clearance, because plasma insulin clearance is greater in glucagon-like peptide 1 (GLP-1) deficient and lower in GLP-1-overexpressing mice and lower after glucose ingestion than during isoglycemic intravenous glucose infusion in people [107,108,109,110,111]. However, these assessments did not take into account the differences in insulin secretion rates in the GLP-1-modified mice and after oral and intravenous glucose administration [112,113], which itself could explain the observed differences in insulin clearance.

## 2. Effects of Obesity and Type 2 Diabetes on Insulin Clearance

### 2.1. Effects of Obesity and T2D on Insulin Receptors and CEACAM 1

Obesity is associated with reduced cell surface insulin receptors in key tissues that are the primary sites of insulin clearance and are also involved in regulating glucose metabolism (including liver, skeletal muscles, and adipocytes) [114,115,116,117,118,119,120,121,122]. In people with obesity and mild insulin resistance, reduced cell surface insulin receptor expression is considered the primary cellular abnormality responsible for insulin resistance whereas in people with severe insulin resistance, post-receptor defects (i.e., defects in insulin signaling and downstream events) also occur [6,117,123,124]. Insulin receptor binding affinity is not reduced in obesity and was sometimes found to be even greater in cells from obese than lean people [115,120,121,122,125]. The decreased cell surface insulin receptor number in people with obesity is thought to be at least in part due to the obesity-associated increase in insulin secretion and concomitant chronic hyperinsulinemia, because receptor number is inversely related to plasma insulin concentration and lowering insulin by administering diazoxide or fasting increased the number of receptors [119,126,127,128,129]. In addition, accelerated net insulin receptor degradation (due to both decreased synthesis and increased breakdown) may also be involved [44,130,131]. The effect of T2D on insulin receptor expression in liver and skeletal muscles has not been extensively studied but the available evidence suggests that there is no cell surface receptor deficit in people with obesity and T2D compared with obese control participants [120,121,132]. Furthermore, studies on fibroblasts demonstrated that the ability of insulin to bind, internalize, and regulate its own receptor is not altered in T2D [133]. This is not unexpected because T2D is primarily due to severe β-cell dysfunction whereas insulin sensitivity is no worse than in people with obesity without T2D [1,3].

Obesity is also associated with reduced hepatic CEACAM 1 expression, and T2D does not alter the relationship between adiposity and CEACAM 1 expression [134,135]. The clinical significance of the decrease in CEACAM 1 on plasma insulin clearance is unclear because of conflicting results from studies that evaluated the functional consequence of altered CEACAM 1 expression. Studies conducted in mice demonstrate reduced CEACAM 1 expression can impair plasma insulin clearance [136,137]. However, the adverse effect of reduced CEACAM 1 expression was only observed in homozygous, but not heterozygous mice [136,137], suggesting only severe CEACAM 1 deficiency, but not more moderate reductions in CEACAM 1 has functional consequences. Furthermore, homozygous mice were much heavier and fatter than heterozygous and wild-type mice and secreted more insulin, consistent with obesity-associated insulin hypersecretion [27]. Overexpression of CEACAM 1 did not alter plasma insulin clearance in chow-fed mice, but blunted the reduction in plasma insulin clearance after high fat diet feeding [138]. It also prevented the high fat diet-induced increase in β-cell mass and insulin secretion, so it is difficult to determine the independent effect of CEACAM 1 overexpression on insulin clearance in high fat diet fed mice.

### 2.2. Effects of Obesity and T2D on Plasma Insulin Clearance

It is well established that people with obesity have higher basal and postprandial plasma insulin concentrations than lean people [4,5,6,9,139]. The mechanisms responsible for the obesity-associated increase in plasma insulin have been a matter of interest for a long time and it has been debated whether hyperinsulinemia is due to insulin hypersecretion or reduced insulin clearance or both, whether the alterations in insulin kinetics are due to excess adiposity per se or secondary to insulin resistance, whether alterations in insulin kinetics are indeed a consequence and not a cause of insulin resistance, and whether increased insulin clearance is a primary cause for the insulin deficiency of T2D [4,7,41,140,141,142,143]. Results from many studies that compared plasma insulin clearance in lean people and people with obesity and those with T2D are inconclusive [76,86,94,144,145,146,147,148,149,150,151]. The discrepancy in results could be due to differences in participant metabolic status. Differences in the methods used to assess insulin clearance may also contribute to the inconsistency in results. Some studies evaluated insulin clearance after intravenous insulin infusion whereas others evaluated insulin clearance during glucose ingestion or during an entire 24 h period. Many of these studies used the C-peptide to insulin concentration ratio as an index of insulin clearance, which has significant limitations [152]. In addition, some studies assessed hepatic insulin extraction without taking into account the contribution of arterial insulin (in addition to insulin secretion) to hepatic insulin delivery whereas others did not take into account the contribution of endogenous insulin secretion during insulin infusion, which can be substantial [14,104,105,106]. In the following sections, we first review the effects of obesity, insulin resistance, and T2D on transendothelial transport (Section 2.2.1). Then (Section 2.2.2, Section 2.2.3, Section 2.2.4, Section 2.2.5), we critically review the results from studies that evaluated the complex relationships among adiposity, insulin sensitivity, insulin secretion, and insulin clearance by using different experimental protocols. We purposely focus on plasma insulin clearance rate, not insulin extraction or uptake rates or other metrics of insulin removal from plasma (unless specifically noted), because insulin clearance rate is the most commonly used metric in these studies. Moreover, we discuss the observed plasma insulin clearance rates in the context of the observed plasma insulin concentrations to provide insight into the dose-dependent and independent effects of obesity and T2D on plasma insulin clearance. We end with a clear perspective based on the findings from several recent studies that simultaneously assessed the effects of obesity, insulin sensitivity, and T2D on plasma insulin clearance and help explain the data from smaller earlier studies.

#### 2.2.1. Transendothelial Insulin Transport

The effects of obesity, insulin resistance, and T2D on transendothelial insulin transport are unclear because few studies have addressed this topic in people. It has been proposed that insulin transport into the interstitium of muscle tissue is slower in people with obesity and insulin resistance compared with lean people, because the rise in muscle microdialysate insulin after intravenous insulin infusion was slower in obese compared with lean participants [153,154]. However, it is not clear whether the slower rise in interstitial insulin was due to impaired transendothelial transport or reduced muscle perfusion, or possibly (though unlikely) even faster interstitial insulin removal. Reduced muscle perfusion during a hyperinsulinemic clamp is often observed in people with obesity, and is due to both capillary rarefaction and impaired insulin-mediated vasodilation [155,156,157,158]. In addition, results from studies conducted in mice suggest that obesity results in ultrastructural alterations to the muscle capillary endothelium which delay endothelial insulin transport [159]. However, these alterations in perfusion and endothelial structure do not affect insulin delivery to myocytes under normal physiological conditions, because the hyperinsulinemia associated with obesity compensates for this defect [159,160,161]. In people with T2D, vascular permeability is increased [162,163] and insulin exchange with the interstitium is enhanced [32].

#### 2.2.2. Plasma Insulin Clearance during Constant Intravenous Insulin Infusion Protocols

Several studies evaluated the relationships among adiposity, insulin sensitivity, and plasma insulin clearance in people with and without T2D by using intravenous insulin infusion protocols (hyperinsulinemic-euglycemic clamp and insulin suppression test) [8,85,86,87,90,91,92,93,94]. In these studies, whole-body insulin clearance was reduced in people with obesity who were insulin resistant compared with both lean people and people with obesity who were insulin sensitive [8,85,87,90,91,92,93]. Furthermore, insulin clearance was not different between lean people and people with obesity who were as insulin sensitive as lean people [93]. Glycemic status and T2D did not affect the relationship between insulin sensitivity and insulin clearance [8]. The results from these studies unanimously demonstrate insulin sensitivity, but not adiposity or dysglycemia, is a determinant of whole-body insulin clearance. The insulin infusion rates during these studies (~30 to 40 mU insulin per m^2^ of body surface area) resulted in arterial insulin concentrations that ranged from approximately 300 pmol/L to <1000 pmol/L [85,91,92,93], which is within the postprandial range [76,77]. However, because of the peripheral administration of insulin, hepatic insulin delivery at any arterial insulin concentration was much lower than at corresponding postprandial arterial insulin concentrations (see Section 1.6. Study protocols to evaluate plasma insulin clearance and their clinical relevance). In addition, peripheral administration of insulin excludes hepatic first pass insulin extraction (see Section 1.6. Study protocols to evaluate plasma insulin clearance and their clinical relevance and Figure 1). One of these studies estimated hepatic insulin clearance and found it was not different between healthy lean participants and participants with T2D who were lean or obese [87]. However, hepatic insulin clearance in this study was calculated by dividing the insulin infusion rate during the hyperinsulinemic clamp procedure by the peripheral plasma insulin concentration during the clamp procedure. This approach erroneously assumes that all of the infused insulin is cleared by the liver; in addition, it does not take into account the delivery of endogenously produced insulin to the liver through the portal vein, which we estimate could contribute as much as 30% to total insulin delivery during the hyperinsulinemic clamp procedure and differ markedly among lean and obese groups [14,104,105,106].

#### 2.2.3. Plasma Insulin Clearance during Glucose Infusion Protocols

Although the assessment of insulin clearance during glucose infusion represents the clearance of endogenously produced insulin, and therefore includes an assessment of hepatic first pass insulin extraction, it is not a good substitute for the assessment of postprandial insulin clearance. Intravenously infused glucose does not stimulate the release of incretins, which potentiate the effect of glucose on β cells [112,113]. Insulin secretion in response to intravenously administered glucose is therefore much less than insulin secretion after glucose ingestion [112,113]. Furthermore. both the incretin response and the incretin effect differ between lean people and people with obesity, insulin resistance, and T2D [112,113,164]. Results from studies that evaluated the relationships among adiposity, insulin sensitivity and plasma insulin clearance by using intravenous glucose infusion protocols suggest that reduced insulin clearance in people with obesity is related to insulin resistance, and is not due to increased body fat per se. Among both lean and obese participants, the overall (area under the curve) insulin clearance rate during constant hyperglycemia or sequential graded glucose infusion with glucose infusion rates ranging from zero to 8 mg/kg body mass/min was less in participants who were insulin resistant compared to those who were insulin sensitive; in addition, insulin clearance correlated positively with insulin sensitivity [76,88,89,90,92]. To our knowledge, the effect of T2D on insulin clearance during glucose infusion has not been evaluated in people. However, results from a study that evaluated insulin clearance during graded glucose infusion in cynomolgus monkeys suggest that T2D is associated with increased insulin clearance [165]. Whether the higher insulin clearance associated with T2D was due to T2D per se or the markedly lower insulin secretion rate in those with T2D is unclear.

#### 2.2.4. Plasma Insulin Clearance during an Oral Glucose Tolerance or Meal Test

**Figure 5:**
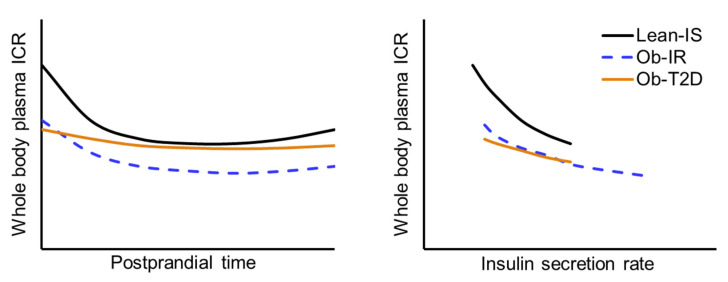
Effect of obesity, insulin resistance, and type 2 diabetes on basal and postprandial plasma insulin clearance rate. Left panel: Whole-body plasma insulin clearance rate during basal conditions and for three hours after glucose ingestion. Right panel: Plasma insulin clearance rate in relationship to insulin delivery to tissues during basal conditions and during the early postprandial period. Adapted from references [3,8,83]. Abbreviations: ICR, insulin clearance rate; IS, insulin sensitive; IR, insulin resistant; Ob, obese; T2D, type 2 diabetes.

Results from studies that included lean participants and participants with obesity with different glycemic status, ranging from normal fasting glucose combined with normal glucose tolerance to those with impaired fasting glucose and/or impaired glucose tolerance and T2D, demonstrate plasma insulin clearance correlates with insulin sensitivity [3,8,166]. They also demonstrate that in people without T2D, plasma insulin clearance and forearm (muscle) insulin fractional extraction decrease rapidly during the first 30 min after glucose ingestion and remain below basal values during the entire two- to three-hour postprandial testing period [8,27,96]. Furthermore, at any insulin secretion rate and plasma insulin concentration, plasma insulin clearance is less in people who are insulin resistant than those who are insulin sensitive [3]. The early decrease in insulin clearance after glucose ingestion is blunted in participants with T2D compared with the respective lean or obese non-diabetic control groups [3,8,96]. However, the postprandial decrease in insulin clearance rate is appropriate for the reduced postprandial insulin secretion and plasma insulin concentration [3] (Figure 5). Plasma insulin clearance remains below basal values even after peak insulin secretion rates have been achieved and insulin secretion rate returns to basal values, presumably because it takes at least 30 min for internalized cell surface insulin receptor recycling (see Section 1.3. Cellular mechanisms of insulin removal from plasma). Furthermore, it was found that intentional weight gain in lean participants until body mass index increased by 2 points caused insulin resistance and decreased plasma insulin clearance during basal conditions and after glucose ingestion, whereas insulin secretion was unchanged [167].

#### 2.2.5. Integrated Multi-Modal Modelling Assessment of Plasma Insulin Clearance

Data obtained from a complex mathematical modelling analysis of plasma insulin clearance rates obtained during a series of different glucose ingestion and intravenous glucose and insulin infusion protocols conducted in 2000 lean and obese men and women suggest that there is an inverse relationship between insulin clearance and insulin secretion rate and insulin sensitivity is by far the most important determinant of plasma insulin clearance at any insulin secretion rate [83]. Plasma insulin clearance decreases as the delivery rate of insulin increases and at any insulin secretion rate, plasma insulin clearance is less in people who are insulin resistant than those who are insulin sensitive [83] (Figure 5).

### 2.3. Non-Alcoholic Fatty Liver Disease and Insulin Clearance in People with Obesity

Non-alcoholic fatty liver disease (NAFLD) is common in people with obesity [139] and associated with insulin resistance [168]. It has been proposed that NAFLD contributes to impaired plasma insulin clearance in people with insulin resistance [169,170,171], presumably because NAFLD impairs hepatic functioning. However, the results from studies that provided a comprehensive analysis of the relationships among insulin secretion in response to glucose ingestion and hepatic, extrahepatic, and whole-body insulin plasma clearance and tissue extraction rates suggest that NAFLD does not impair hepatic insulin extraction per se, but rather the lower hepatic and whole-body insulin clearance in people with NAFLD are due to insulin resistance and insulin hypersecretion [84,95]. A study that used Mendelian Randomization analysis to evaluate the relationship between NAFLD and plasma insulin clearance based on genetics, also found no support for a causal link between hepatic steatosis and hepatic insulin clearance [172].

## 3. Summary and Conclusions

Plasma insulin clearance is a highly dynamic, receptor-mediated process and an important determinant of plasma insulin concentration. Basal insulin receptor expression and insulin delivery to tissues that clear insulin are key determinants of insulin clearance. Insulin sensitivity correlates with plasma insulin clearance, presumably because both insulin action in and insulin clearance by tissues require insulin binding to its receptors and insulin receptor expression is downregulated in people with insulin resistance. Moreover, insulin clearance is inversely related to the insulin delivery rate to tissues, so the postprandial increase in insulin secretion itself causes a decrease in insulin clearance and chronic hyperinsulinemia is at least in part responsible for reduced cell surface insulin receptor expression in people with obesity. The higher postprandial insulin clearance in people with T2D, compared to those without, is a consequence of β-cell dysfunction and reduced insulin secretion, which blunts the postprandial insulin delivery rate to tissues that clear insulin. The greater insulin clearance in people with T2D is due to impaired β-cell function, which blunts the postprandial downregulation of insulin clearance caused by insulin receptor internalization after insulin binding.
